# Rotavirus-Associated Hospitalization in Children With Subsequent Autoimmune Disease

**DOI:** 10.1001/jamanetworkopen.2023.24532

**Published:** 2023-07-26

**Authors:** Eun Kyo Ha, Ju Hee Kim, Hye Ryeong Cha, Gi Chun Lee, Jeewon Shin, Youn Ho Shin, Hey-Sung Baek, Seung Won Lee, Man Yong Han

**Affiliations:** 1 Department of Pediatrics, Hallym University Kangnam Sacred Heart Hospital, Seoul, Korea; 2 Department of Pediatrics, Kyung Hee University Hospital, Kyung Hee University School of Medicine, Seoul, Korea; 3 Sungkyunkwan University School of Medicine, Suwon, Korea; 4 School of Computer Science and Engineering, Konkuk University, Seoul, Korea; 5 Department of Pediatrics, Bundang CHA Medical Center, CHA University School of Medicine, Seongnam, Korea; 6 Center for Digital Health, Medical Science Research Institute, Kyung Hee University Medical Center, Kyung Hee University College of Medicine, Seoul, Korea; 7 Department of Pediatrics, Hallym University Kangdong Sacred Heart Hospital, Seoul, Korea

## Abstract

**Importance:**

Rotavirus infection is a common cause of gastroenteritis in children that can trigger autoimmune processes, but the nature of this interaction remains poorly understood.

**Objective:**

To estimate the association of rotavirus infection with the risk of subsequent autoimmune disease.

**Design, Setting, and Participants:**

This population-matched cohort study used data from children and adolescents (aged younger than 18 years) in South Korea (national registers from January 1, 2002, to December 31, 2017). The cohort consisted of 86 157 patients in the exposure group who had experienced rotavirus-associated hospitalization and the same number of matched patients in the unexposed group. Data analyses were from May 1, 2020, through October 20, 2022.

**Exposures:**

Hospitalization for rotavirus infection.

**Main Outcomes and Measures:**

The main outcome was childhood autoimmune diseases during the study defined by diagnoses according to the National Health Insurance Database. Hazard ratios (HRs) with 95% CIs for autoimmune diseases were estimated using a Cox model, with multiple confounding factors controlled.

**Results:**

This cohort study consisted of 1 914 461 individuals born in South Korea from 2002 to 2005 who were potentially eligible. After exclusions, there were 86 517 individuals in the exposed group and 86 517 in the unexposed group after 1:1 incidence density sampling. The study included 49 072 (57.0%) male patients. The median (IQR) age at diagnosis of rotavirus-associated hospitalization was 1.5 (0.9-2.7) years, and the HR for autoimmune disease in the exposed group was 1.24 (95% CI, 1.19-1.28) for a mean (SD) follow-up time of 12.1 (3.2) years. The use of more stringent definitions for exposure and outcomes in a multivariable stratified analysis also indicated that rotavirus-associated hospitalization was associated with an increased risk of subsequent autoimmune disease (HR, 1.22 [95% CI, 1.16-1.28]). Sensitivity analysis showed that individuals with rotavirus-associated hospitalization was related to multiple autoimmune syndromes (2 or more: HR, 1. 51 [95% CI, 1. 31-1. 73]; 3 or more: HR, 1. 79 [95% CI, 1.18-2.72]) and that the number of rotavirus-associated hospitalization were associated with higher risks for autoimmune disease in a dose-dependent manner (single hospitalization event: HR, 1.20 [95% CI, 1.16-1.24]; multiple events HR, 1.60 [95% CI, 1.49-1.72]).

**Conclusions and Relevance:**

Our results indicate that rotavirus-associated hospitalization is significantly associated with subsequent autoimmune disease during childhood. Clinicians should be aware of the heightened susceptibility to autoimmune disease in individuals with prior rotavirus-associated hospitalization.

## Key Points

**Question:**

Rotavirus infection is a common cause of gastroenteritis in children, but is it associated with further autoimmune processes?

**Findings:**

In this cohort study of nearly 2 million Korean children, the occurrence of rotavirus-associated hospitalization was found to be associated with a 1.24-fold higher risk of autoimmune disease compared with controls.

**Meaning:**

Rotavirus-associated hospitalizations were significantly associated with risk of subsequent autoimmune disease.

## Teaser

This cohort study assesses the association of rotavirus infection with the risk of subsequent autoimmune disease in children and adolescents in South Korea.

## Introduction

Rotavirus infection is a leading cause of mortality and morbidity in infants and young children, and these infections are also linked to autoimmunity.^1,2,3^ While some individuals recover without complications, others develop manifestations affecting various organs, and viral dissemination may occur.^4,5,6,7,8^ Moreover, some infected children experience extraintestinal complications that influence specific organs or whole body systems. Several lines of evidence suggest that rotavirus infection can trigger autoimmune diseases.

There are 9 species of rotavirus, each with a genome comprising double-stranded RNA segments encoding structural and nonstructural proteins. Vaccinations have reduced the prevalence of rotavirus disease, but postinfection illnesses persist. There is also evidence linking rotavirus infection to autoimmune diseases, such as celiac disease,^4,6,9^ type 1 diabetes,^7,10,11^ pancreatitis,^12^ and neuropathy.^13,14^ However, previous studies had limitations in terms of controls,^4,6,11^ small sample sizes,^12,13,14^ and study design.^7,10,13^ While there is supporting evidence from humans and animals, clinical epidemiology studies have only provided limited support. We performed a systematic literature search and analyzed a nationwide population-based database of nearly 2 million individuals to investigate the association between rotavirus-associated hospitalization and subsequent autoimmune disease, while accounting for confounding factors.

## Methods

The study received approval from the institutional review board and ethics committees of Hallym University Kangnam Sacred Heart Hospital and the Korea National Institute for Bioethics Policy. Informed consent was waived by these committees because the study used deidentified data that were publicly available in Korea. We followed the Reporting of Studies Conducted Using Observational Routinely-Collected Data (RECORD).

### Systematic Search and Identification of Published Literature

We performed a literature search in PubMed using the following Medical Subject Heading (MeSH) terms: rotavirus, rotavirus infection, and autoimmune disease. All relevant studies conducted after 1999 were eligible for inclusion in the review. Studies were included if they (1) examined the incidence of any autoimmune disease–related hospitalizations or outpatient visits after rotavirus infection; (2) obtained fecal samples to test for rotavirus in patients with autoimmune diseases; and (3) examined the distribution of specific types of rotavirus related to autoimmunity in vivo or in vitro. We included all studies conducted among children up to 18 years of age. Editorials, narrative, or subject reviews, and symposium proceedings were excluded. Studies that investigated rotavirus vaccination or were conducted before 1990 were excluded.

The literature search yielded 338 citations with relevant keywords for rotavirus infection and specific autoimmune diseases; of these, 85 evaluated the burden of any autoimmune diseases or rotavirus strain linked autoimmunity. Of the 30 studies included in this literature search, 26 were human studies reporting rotavirus-associated autoimmune diseases or autoimmunity (eTable 1 in Supplement 1) and 4 studies were nonhuman studies (eTable 2 in Supplement 1). Rotavirus infection was related to various groups of autoimmune diseases, including diseases of the digestive system (eg, inflammatory bowel disease,^20^ celiac disease,^4,6^ nonceliac gluten sensitivity [NCGS],^3^ pancreatitis^21^) and endocrine system (eg, type 1 diabetes^11,21,22^). Associations for risk of other diseases including the nervous system (eg, Guillain-Barre syndrome,^23^ acute [necrotizing] encephalopathy,^24,25^ and opsoclonus-myoclonus syndrome^26^), skin (eg, pemphigus vulgaris^27^), and vasculitis (eg, immune thrombocytopenic purpura,^28^ Kawasaki disease,^29^ and hemolytic uremic syndrome^30^) were also documented.

### Design for the Cohort Study

**Figure 1.**
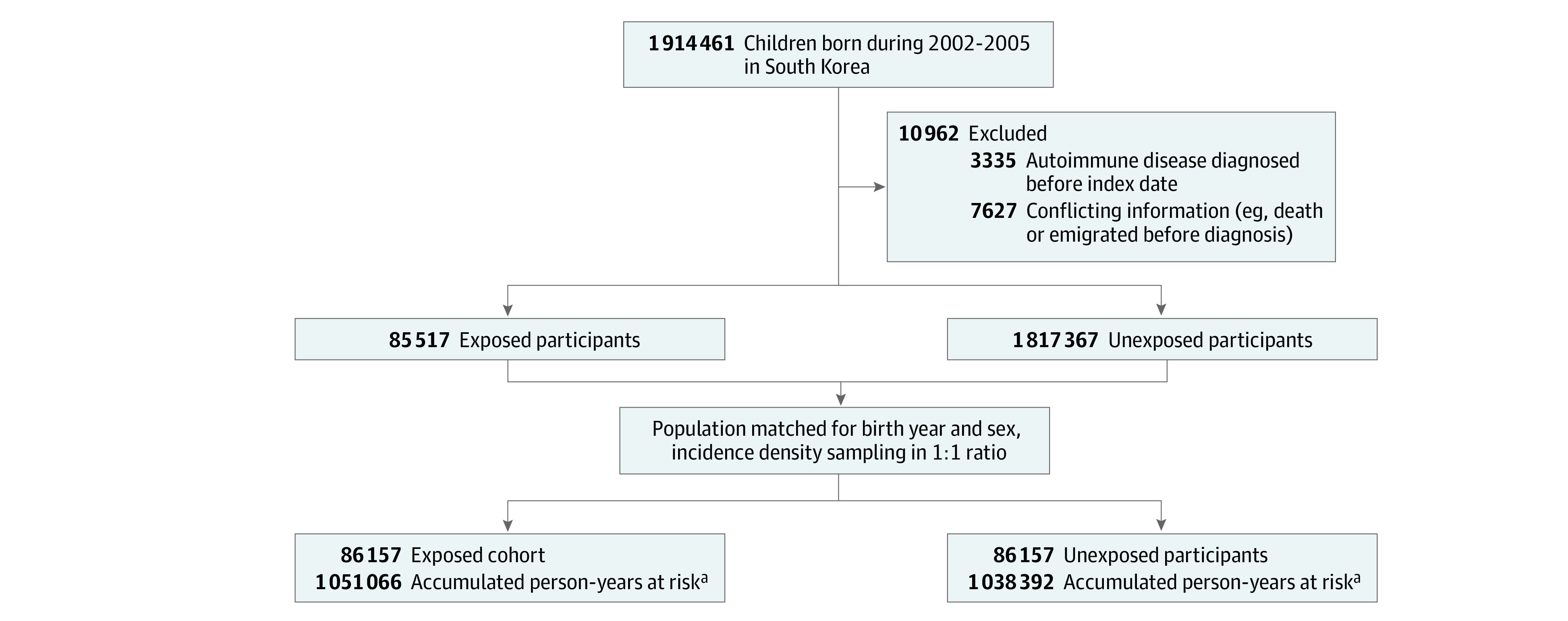
Study Design After application of exclusion criteria, there were 49 937 children in the exposed cohort and 1 277 613 matched children in the unexposed cohort. ^a^The first year of follow-up was excluded for calculation of accumulated person-years.

Individuals born in South Korea between 2002 and 2005 were identified in the National Health Insurance Service (NHIS) database and linked information on deaths from Statistics Korea.^15^ Data analyses were from May 1, 2020, to October 20, 2022. NHIS is a single medical insurer that covers approximately 98% of South Korea’s population that predominantly consists of Korean nationals. All medical institutions submit health care use-related data to ensure reimbursement, which are stored in the NHIS database. This database includes claims data, information on the demographics, health care use (using the *International Statistical Classification of Diseases and Related Health Problems, Tenth Revision *[*ICD-10*]), prescriptions, and procedures. Children who required hospitalization due to a rotavirus infection were defined as the exposed group (Figure 1). The date of the diagnosis was set as the index date, and all included participants were younger than 19 years on that date. Patients were excluded if they had a history of autoimmune disease before the index date or had records with conflicting information. The exposed group was then compared with a population-matched unexposed group that was established using 1:1 matching by density sampling. Each participant in the unexposed group was randomly and individually matched by birth year and sex, and had no history of rotavirus-associated hospitalization or autoimmune disease at the date of diagnosis of the index patient.

### Exposure

The specific exposure of interest was hospitalization for rotavirus infection as the main or secondary diagnosis, identified using an *ICD-10* code of A08.0X (rotavirus enteritis) (eTable 3 in Supplement 1). The diagnosis of rotavirus infection with hospitalization was based on previous studies and established guidelines. A previous study reported the positive predictive value (PPV) for this rotavirus code was 96.8% (95 CI, 94.8%-98.3%), indicating high validity.^16^

### Follow-Up

All participants were followed up from the index date until the first diagnosis of an autoimmune disease, death, or the end of the study (December 31, 2017), whichever happened first. The follow-up of individuals in the unexposed group involved assessing rotavirus-associated hospitalization at a later time. If an individual experienced a rotavirus-associated hospitalization during the follow-up period, they were censored and transferred to the exposed group. The first year of follow-up was excluded from the analyses to reduce the probabilities of reverse causality and surveillance bias.

### Autoimmune Diseases

Information on autoimmune diseases was from the NHIS database and corresponding *ICD-10* codes (eTable 3 in Supplement 1).^17^ In total, 41 autoimmune diseases were considered because previous studies suggested these were related to rotavirus infection. The outcome and time frame were based on previous human and nonhuman studies (eTables 1, 2, 4, and 5 in Supplement 1).

### Covariates

Data regarding household income, birth residence, season of birth, and perinatal status (eg, disorder related to length of gestation and fetal growth [*ICD-10*: P05X to P08X]; birth trauma [*ICD-10*, P10X to P15X]; infections specific to the perinatal period [*ICD-10*, P35X to P39X]; and congenital malformations, deformations, and chromosomal abnormalities [*ICD-10*, Q00X to Q99X]) were examined.^15^ The analysis considered the latest available information before the index date. Medical resource usage, including inpatient and outpatient visits, was collected during follow-up. Additionally, antibiotic, systemic steroid use, and hospitalization duration were assessed.

### Statistical Analysis

We used a conditional Cox proportional hazard model to estimate hazard ratios (HRs) and 95% CIs of autoimmune disease in relation to rotavirus-associated hospitalization using time after the index date as the underlying time scale. The visual inspection of covariates using Schoenfeld residuals in the Cox proportional hazard regression indicated no violations of the assumption. The analysis did not examine a specific autoimmune disease with fewer than 100 cases separately, but incorporated it in the HR calculations for its corresponding major group. All analyses were stratified by matching identifiers and adjusted for birth residence, household income, and perinatal history. HRs were separately calculated for sex, age at the index date (24 months or less or more than 24 months), calendar year of birth (2002-2003 or 2004-2005), calendar year of birth at the index date (2002-2009 or 2010-2017), birth residence (Seoul/metropolitan, city, or rural area), household income (low or high), season of birth (spring, summer, fall, or winter), disorders related to any perinatal status (yes or no), history of asthma (yes or no), history of hospital admission (yes or no), and number of outpatient visits (15 or fewer or more than 15, based on the median) during the first year after study entry. Data collected during the first month after study entry were not considered, because participants in the exposed group used more health care services during the first month after diagnosis of infection. The first year of follow-up was excluded from all analyses. Differences in HRs were assessed by introducing an interaction term into the Cox models. The absolute rate differences and 95% CIs were determined for all associations. The difference in log (HR) between strata was used to calculate the *z* score and *P* value for this comparison. We examined the risk of a diagnosis of any autoimmune disease as 9 major groups of autoimmune diseases (inflammatory arthritis; vasculitis; connective tissue disorders; and diseases of the digestive, endocrine, skin, hematologic, and nervous systems, among others).

Sensitivity analyses were performed to examine the association using a more stringent definition of the exposure (rotavirus enteritis as the main diagnosis of hospitalizations were included in the exposure group, and cases of rotavirus enteritis as a secondary diagnosis were not considered as the exposure group), 2 or more autoimmune syndromes or 3 or more autoimmune syndromes, individual autoimmune diseases, any of the major groups of autoimmune diseases (eTable 2 in Supplement 1),^17^ and a more stringent definition of outcome (*ICD-10* code and use of nonsteroidal anti-inflammatory drug, systemic steroid, intravenous immunoglobulin,^18,19^ or thyroid medication). Additionally, the relationship of the total number of hospitalizations and duration of hospitalization were also analyzed. Taking into account possible surveillance bias and reverse causality, it is plausible that patients hospitalized for rotavirus infection may have had preexisting autoimmune conditions, and rotavirus infection could have occurred as a complication of hospital stays related to autoimmune diseases or other infections. To address this, the analyses were repeated by excluding participants who were enrolled during the first 2 years (2002-2003) or during the first 5 years (2002-2007). In these analyses, the cumulative incidence curves of autoimmune diseases among participants with more than 1 year of follow-up were analyzed. All statistical analyses were conducted using SAS version 9.4 (SAS Institute), and a 2-sided *P* < .05 was considered statistically significant.

## Results

This cohort study consisted of 1 914 461 individuals born in South Korea from 2002 to 2005 who were potentially eligible. We excluded 10 962 of these individuals, due to diagnoses of autoimmune diseases before the index date (3335 [30.4%]) or conflicting data (7627 [69.6%]) (Figure 1). Thus, there were 86 517 individuals in the exposed group, and the same number in the unexposed group after 1:1 incidence density sampling. The median (IQR) age at rotavirus-associated hospitalization was 1.5 (0.92-2.67) years and 49 072 (57.0%) of all patients were male (Table 1). Compared with the unexposed group, the exposed group had more inpatient and outpatient hospital visits during the first year after study entry and had increased use of prescription medications, especially antibiotics and systemic steroids. During the mean (SD) follow-up time of 12.05 years (3.17), we identified newly diagnosed autoimmune diseases in 7589 of 86 517 individuals (8.8%) in the exposed group (incidence rate, 73.1 per 10 000 person-years) and in 6095 of 86 517 individuals (7.0%) in the unexposed group (incidence rate, 58.0 per 10 000 person-years), corresponding to an absolute difference of 15.1 per 10 000 person years (95% CI, 12.90-17.29 per 10 000 person-years).

**Table 1.** Characteristics of the Study Cohorts

Characteristic	Population-matched cohort, No. (%), (N = 86 157)	
	Exposed group	Matched unexposed group
Follow-up time, mean (SD), y	12.05 (3.17)	12.20 (2.98)
Sex		
Female	37 085 (43.0)	37 085 (43.0)
Male	49 072 (57.0)	49 072 (57.0)
Age at index date, median (IQR), y	1.50 (0.92-2.67)	1.50 (0.92-2.67)
Age group, mo		
≤60	54 485 (63.2)	54 485 (63.2)
>60	31 672 (36.8)	31 672 (36.8)
Birth residence^a^		
Seoul/Metropolitan	45 644 (53.0)	45 445 (52.8)
City	31 043 (36.0)	32 050 (37.2)
Rural	9469 (11.0)	8649 (10.0)
Household income^b^		
Low (≤median)	24 774 (28.8)	21 847 (26.3)
High (>median)	58 855 (68.3)	61 076 (73.7)
Calendar year at birth		
2002-2003	37 582 (43.6)	37 582 (43.6)
2004-2005	48 575 (56.4)	48 575 (56.4)
Calendar season of birth		
Spring	21 757 (25.3)	22 525 (26.1)
Summer	20 724 (24.1)	20 184 (23.4)
Fall	22 567 (26.2)	21 436 (24.9)
Winter	21 109 (24.5)	22 012 (25.5)
Inpatient hospital visits during the first year after study entry^c^		
None	59 740 (69.3)	75 410 (87.5)
≥1	26 417 (30.7)	10 747 (12.5)
Any perinatal status		
Yes	22 582 (26.2)	16 683 (19.4)
No	63 575 (73.8)	69 474 (80.6)
Fetal growth and development disorder^d^		
Yes	1619 (1.9)	805 (0.9)
No	84 538 (98.1)	85 352 (99.1)
History of birth injury		
Yes	272 (0.3)	156 (0.2)
No	85 885 (99.7)	86 001 (99.8)
Infections specific to the perinatal period		
Yes	7679 (8.9)	5062 (5.9)
No	78 478 (91.1)	81 095 (94.1)
Congenital malformation		
Yes	1995 (2.3)	1320 (1.5)
No	84 162 (97.7)	84 837 (98.5)
Any intestinal infectious disease and admission, before 6 mos prior to the index date^e^		
Yes	9383 (10.9)	5600 (6.5)
No	76 774 (89.1)	80 557 (93.5)
Use of antibiotics^f^		
Yes	68 149 (79.1)	12 947 (15.0)
No	18 008 (20.9)	73 210 (85.0)
Use of systemic corticosteroids^f^		
Yes	8979 (10.4)	1804 (2.1)
No	77 178 (89.6)	84 353 (97.9)
ICU care during a month after index date^g^		
Yes	887 (1.0)	NA
No	85 270 (99.0)	NA
Length of hospitalization, d^h^		
≤5	45 400 (52.7)	NA
>5	40 757 (47.3)	NA
Outpatient hospital visits during the first year after study entry		
≤15	38 652 (44.9)	53 456 (62.0)
>15	47 505 (55.1)	32 701 (38.0)

Abbreviations: ICU, intensive care unit; NA, not available.

^a^ Missing information in weighted data: control group, 1; exposed group, 13.

^b^ Income was classified into 2 groups based on the regional median income. Missing information in weighted data: control group, 2528; exposed group, 3234.

^c^ The first month after study entry was removed from this calculation.

^d^ Complications during the intrauterine and perinatal period that were diagnosed 3 months prior to the index date (ie, diagnosis date of the exposed participant or the diagnosis date of the index participant for the matched unexposed participant).

^e^ Defined as one or more physician diagnosis during 6 months prior to the index date requiring hospitalization with a main diagnosis of intestinal infectious disease (A00X to A09X) other than rotavirus infection (A08.0X).

^f^ Prescriptions during the month after the index date.

^g^ Events during 1 month after the index date.

^h^ The median length of hospitalization was 5 days.

**Figure 2.**
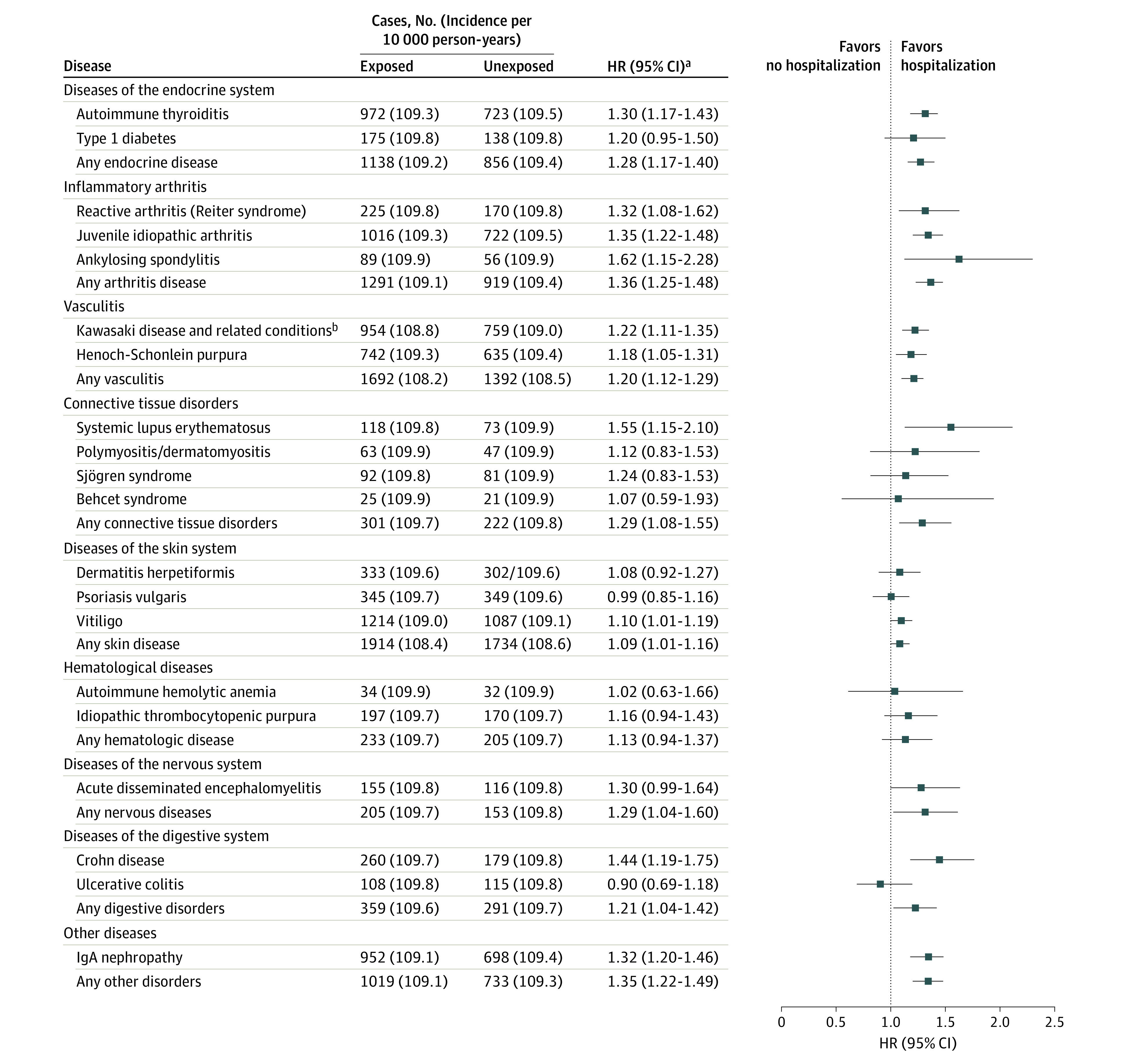
Risk Estimates for the Association of Rotavirus-Associated Hospitalization With Different Types of Autoimmune Diseases Cox models were stratified by matching identifiers (birth year and sex) and adjusted for birth residence (Seoul/metropolitan, city, or rural area), household income (low, middle, or high), and perinatal history (disorder related to length of gestation and fetal growth, birth trauma, infections specific to the perinatal period, congenital malformation/deformation, and chromosomal abnormalities). The first year of follow-up was excluded from all analyses. HR indicates hazard ratio; IgA, immunoglobin A. ^a^Autoimmune diseases with fewer than 100 cases were not analyzed separately, but were included in calculations of HRs for the main categories. ^b^Includes autoimmune vascular disorders, such as polyarteritis nodosa and Churg-Strauss syndrome.

After controlling for confounders, the exposed group had a significantly greater risk for autoimmune disease (HR, 1.24; 95% CI, 1.19-1.28) (Table 2). Rotavirus-associated hospitalization was associated with higher rates for each of the 9 major groups of autoimmune diseases (Figure 2). The population-matched analysis indicated these associations were notable for the following groups: inflammatory arthritis (HR, 1.36; 95% CI, 1.25-1.48), connective tissue disorders (HR, 1.29; 95% CI, 1.08-1.55), nervous system diseases (HR, 1.29; 95% CI, 1.04-1.60), endocrine diseases (HR, 1.28; 95% CI, 1.17-1.40), and vasculitis (HR, 1.20; 95% CI, 1.12-1.29). In agreement, the Kaplan-Meier cumulative incidence curves showed that rotavirus-associated hospitalized individuals were more likely to receive a diagnosis of an autoimmune disease (eFigure in Supplement 1).

**Table 2.** Risk of Autoimmune Disease in the Exposed Cohort (Rotavirus Infection) Relative to the Matched Unexposed Cohort

Characteristic	Patients admitted with rotavirus infection^a^				
	Cases/accumulated person-years ×10 000 (Incidence rate/10 000 person-years)		Absolute rate difference/10 000 person-years (95% CI)	Hazard ratio (95% CI)^b^	*P* value^c^
	Exposed group	Matched unexposed group			
All	7589/ 86 157 (73.1)	6095/86 157 (58.0)	15.10 (12.90 to 17.29)	1.24 (1.19 to 1.28)	
Sex					
Female	3434/37 085 (77.27)	2668/37 085 (59.14)	18.13 (14.71 to 21.55)	1.29 (1.22 to 1.36)	.05
Male	4155/49 072 (69.95)	3427/49 072 (57.12)	12.82 (9.96 to 15.68)	1.20 (1.14 to 1.25)	
By age at index date, mos					
≤24	5335/54 485 (74.78)	4332/54 485 (59.96)	14.82 (12.14 to 17.51)	1.22 (1.18 to 1.28)	.36
>24	2254/31 672 (69.37)	1763/31 672 (53.66)	15.72 (11.92 to 19.53)	1.27 (1.19 to 1.35)	
Time since index date, y^d^					
1 to <5	7224/78 383 (73.46)	5806/78 383 (58.30)	15.15 (12.89 to 17.42)	1.24 (1.19 to 1.28)	.88
≥5	365/7774 (66.36)	289/7774 (52.31)	14.10 (5.01 to 23.19)	1.25 (1.07 to 1.46)	
By calendar year at index date^e^					
2002-2009	7324/80 026 (73.43)	5898/80 202 (58.30)	15.12 (12.87 to 17.37)	1.24 (1.19 to 1.28)	.62
2010-2017	265/6131 (64.76)	197/5955 (49.93)	14.92 (4.46 to 55.38)	1.29 (1.07 to 1.56)	
By calendar year of birth					
2002-2003	3604/37 582 (80.59)	2780/37 582 (61.30)	19.29 (15.81 to 22.77)	1.29 (1.22 to 1.35)	.04
2004-2005	3985/48 575 (67.41)	3315/48 575 (55.47)	11.93 (9.11 to 14.75)	1.19 (1.14 to 1.25)	
Birth residence					
Seoul and metropolitan	4250/45 644 (77.45)	3318/45 445 (59.87)	17.60 (14.49 to 20.68)	1.26 (1.21 to 1.32)	.25
City	2552/31 043 (67.92)	2168/32 050 (55.44)	12.48 (8.96 to 16.00)	1.20 (1.13 to 1.27)	
Rural	787/9469 (69.09)	609/8649 (57.62)	11.48 (4.83 to 18.13)	1.20 (1.08 to 1.35)	
Household income^f^					
Low (≤median)	2163/24 774 (72.68)	1519/21 847 (57.00)	15.68 (11.49 to 19.88)	1.26 (1.18 to 1.34)	.61
High (>median)	5210/58 855 (73.32)	4374/61 076 (58.70)	14.61 (11.97 to 17.26)	1.23 (1.18 to 1.28)	
Calendar season at birth date					
Spring	1984/21 757 (75.86)	1668/22 525 (61.02)	14.86 (10.42 to 19.30)	1.21 (1.13 to 1.29)	.95
Summer	1820/20 724 (73.06)	1397/20 184 (56.61)	16.47 (11.98 to 20.94)	1.26 (1.171 to 1.351)	
Fall	1938/22 567 (70.96)	1440/21 436 (54.96)	16.00 (11.75 to 20.25)	1.28 (1.191 to 1.369)	
Winter	1847/21 109 (72.54)	1590/22 012 (59.13)	13.42 (9.01 to 17.82)	1.21 (1.129 to 1.295)	
Any medical condition during the perinatal period^g^					
Yes	2294/22 582 (83.66)	1431/16 683 (71.10)	12.57 (7.55 to 17.60)	1.19 (1.109 to 1.269)	.17
No	5295/63 575 (69.29)	4664/69 474 (54.88)	14.40 (11.96 to 16.85)	1.25 (1.205 to 1.305)	
Disorders related to length of gestation and fetal growth (P05X to P08X)^g^					
Yes	145/1619 (70.79)	72/805 (75.21)	−4.27 (−25.07 to 16.53)	0.95 (0.74 to 1.26)	.06
No	7444/84 538 (73.13)	6023/85 352 (57.83)	15.30 (13.09 to 17.51)	1.24 (1.20 to 1.28)	
Birth injury (P10X to P15X)^g^					
Yes	21/272 (60.54)	13/156 (68.67)	−8.43 (−53.61 to 36.77)	1.01 (0.50 to 2.05)	.34
No	7568/85 885 (73.13)	6082/86 001 (57.97)	15.16 (12.96 to 17.36)	1.24 (1.20 to 1.28)	
Infections speficific to the perinatal period (P35X to P39X)^g^					
Yes	751/7679 (78.57)	404/5062 (65.54)	12.97 (4.46 to 21.48)	1.20 (1.06 to 1.35)	.55
No	6838/78 478 (72.53)	5691/81 095 (57.52)	15.01 (12.73 to 17.29)	1.24 (1.20 to 1.29)	
Digestive system disorders of fetus and newborn (P75 to P78X)^g^					
Yes	175/1995 (70.70)	112/1320 (70.25)	0.12 (−16.59 to 16.84)	1.05 (.82 to 1.33)	.13
No	7414/84 162 (73.14)	5983/84 837 (57.80)	15.34 (13.13 to 17.56)	1.24 (1.20 to 1.28)	
Congenital malformations (Q00X to Q99X)^g^					
Yes	1600/14 825 (90.02)	1020/11 311 (75.00)	15.04 (8.66 to 21.42)	1.20 (1.10 to 1.30)	.41
No	5989/71 332 (69.59)	5075/74 846 (55.46)	14.12 (11.79 to 16.46)	1.25 (1.20 to 1.29)	
Outpatient hospital visits during the first year after study entry^h^					
>15	5002/53 456 (77.36)	3095/38 652 (65.68)	14.46 (11.43 to 17.50)	1.25 (1.19 to 1.32)	.01
≤15	2587/32 701 (66.08)	3000/47 505 (51.74)	10.53 (7.20 to 13.87)	1.14 (1.09 to 1.20)	
Hospital admission during the first year after study entry^h^					
Yes	4891/59 740 (68.33)	5088/75 410 (55.51)	12.82 (10.37 to 15.27)	1.21 (1.16 to 1.26)	.04
No	2698/26 417 (83.63)	1007/10 747 (74.91)	8.71 (3.11 to 14.31)	1.11 (1.03 to 1.19)	

^a^ Results not shown if the number of participants was less than 10.

^b^ Cox models were stratified by matching identifiers (birth year and sex) and adjusted for birth residence (Seoul/metropolitan, city, or rural area), household income (low, middle, or high), and perinatal history (disorder related to length of gestation and fetal growth, birth trauma, infections specific to the perinatal period, congenital malformation/deformation, and chromosomal abnormalities). The first year of follow-up was excluded from all analyses.

^c^ *P* value was from an interaction test by incorporating an interaction term into the Cox model, which included the covariates and their interaction with the exposure group.

^d^ Complications during the intrauterine and perinatal period that was diagnosed 3 months prior to the index date (ie, the date of diagnosis of a participant in the exposed group or the date of diagnosis of the index participant with the matched participant in the unexposed group). History of fetal growth and development disorders, birth injury, infections specific to the perinatal period, and congenital malformations.

^e^ The calendar year at the index date was divided into 2 groups based on the changing prevalence trend of rotavirus infection following the introduction of rotavirus vaccination in Korea.

^f^ Estimated by insurance copayment amount.

^g^ Conditions that originated in the perinatal period identified by *International Statistical Classification of Diseases and Related Health Problems, Tenth Revision* codes.

^h^ The first month after study entry was removed from this calculation.

Calendar year of birth, calendar season of the birth date, and perinatal status had no significant associations with the HR for autoimmune disease (Table 2). However, the HR was lower in males than females (HR, 1.20; 95% CI, 1.14-1.25 vs HR, 1.29; 95% CI, 1.22-1.36; *P* for interaction = .05). Additionally, it was higher in patients with more outpatient hospital visits during the first year after the study entry (HR, 1.25; 95% CI, 1.19-1.32 vs HR, 1.14; 95% CI, 1.09-1.20; *P* for interaction = .51), although the difference was not statistically significant, as well as those with a history of hospital admission during the first year after study entry (HR, 1.21; 95% CI, 1.16-1.26 vs HR, 1.11; 95% CI, 1.103-1.19; *P* for interaction = .04). There were no significant associations with other indicators of rotavirus infection (use of antibiotics or systemic steroids and frequency of outpatient visits within 1 month after the index date) on the risk of autoimmune disease (eTable 6 in Supplement 1).

We performed a sensitivity analysis to examine the robustness of the results from the primary analysis (Table 3). The results indicated the association of rotavirus-associated hospitalization with autoimmune disease was stronger for those with 2 or more autoimmune syndromes (HR, 1.51; 95% CI, 1.31-1.73) and with 3 or more autoimmune syndromes (HR, 1.79; 95% CI, 1.18-2.72). This sensitivity analysis also indicated greater risk when excluding cases diagnosed 2 years after study entry or 5 years after study entry, when exposure was defined more stringently (rotavirus enteritis as the main diagnosis of hospitalization), and when the outcome was defined by the diagnosis of an autoimmune disease and the use of medication for it. There was an increased risk of autoimmune disease in patients depending on the hospitalized duration (5 days or less: HR, 1.17; 95% CI, 1.12-1.22; more than 5 days: HR, 1.31; 95% CI, 1.26-1.37; *P* for interaction = .04) (eTable 7 in Supplement 1) and number of hospitalization events (single event: HR, 1.20; 95% CI, 1.16-1.24; multiple events: HR, 1.60; 95% CI, 1.49-1.72; *P* for interaction <.001) (eTable 7 in Supplement 1 ).

**Table 3.** Sensitivity Analysis of the Association of Rotavirus Infection With Autoimmune Disease

Sensitivity analysis (reference group)	Patients admitted with Rotavirus infection			
	Cases/accumulated person-years ×10 000 (Incidence rate/10 000 person-years)		Absolute rate difference/10 000 person-years (95% CI)	Hazard ratio (95% CI)^a^
	Exposed individuals	Matched unexposed individuals		
Multiple autoimmune syndromes (any syndrome)^b^				
≥2	245/6.9 (35.5)	1619/70 (23.1)	11.96 (7.42-16.50)	1.51 (1.31-1.73)
≥3	26/7.0 (3.7)	150/70 (2.1)	1.54 (0.99-2.09)	1.79 (1.18-2.72)
Lag-time^c^				
2-y after study entry was excluded	6843/96.2 (71.1)	5549/97 (57.1)	14.03 (11.77-16.28)	1.22 (1.18-1.27)
5-y after study entry was excluded	4902/72.8 (67.4)	4022/73 (54.9)	12.46 (9.93-15.00)	1.20 (1.15-1.26)
Restricted exposure^d^				
Rotavirus infection	3942/56 (70.4)	3233/57(57.2)	13.29 (10.34-16.25)	1.22 (1.16-1.28)
More stringent definition of the outcome^e^				
Diagnosis and medication	687/23.4 (29.3)	5360/235 (22.8)	6.53 (4.25-8.80)	1.27 (1.18-1.38)

^a^ Cox models were stratified by matching identifiers (birth year and sex) and adjusted for birth residence (Seoul/metropolitan, city, or rural area), household income (low, middle, or high), and perinatal history (disorder related to length of gestation and fetal growth, birth trauma, infections specific to the perinatal period, congenital malformation/deformation, and chromosomal abnormalities). The first year of follow-up was excluded from all analyses.

^b^ Coexistence of multiple autoimmune diseases.

^c^ Sensitivity analysis was performed using extension of the lag-time period.

^d^ Rotavirus enteritis as the main diagnosis were included in the exposure group; cases of rotavirus enteritis as a secondary diagnosis were not considered as the exposure group.

^e^ *International Statistical Classification of Diseases and Related Health Problems, Tenth Revision* code of autoimmune diseases and use of immunomodulatory medications (nonsteroidal anti-inflammatory drug, systemic steroid, intravenous immunoglobulin), or thyroid medication.

## Discussion

The results of this large nationwide cohort study of Korean children demonstrated that exposure to rotavirus-associated hospitalization increased the risk for subsequent autoimmune disease. This association remained significant after further sensitivity analysis, in groups with different demographic and perinatal status, and in subgroup analysis that considered medication records. Additionally, our findings demonstrated that patients with longer hospitalization and more frequent hospitalization events due to rotavirus infection had increased risk of autoimmune disease. To the best of our knowledge, this is the first study to examine the association of rotavirus infection with major autoimmune diseases in children using a population-based comparison.

Associations differed among various individual autoimmune diseases and groups of autoimmune diseases. For example, the HR was 1.28 (95% CI, 1.17-1.40) for any endocrine disease and 1.36 (95% CI, 1.25-1.48) for any arthritic disease. The differences may be attributed to mechanistic interactions between autoimmune diseases and rotavirus infection and variances in pathogenicity.

Our results support previous studies linking rotavirus-induced autoimmunity to arthritis, vasculitis, and neurologic diseases.^31,32^ Previous studies indicated that rotavirus infection could potentially trigger autoimmunity in immune-privileged sites,^2^ including the intestine,^3,6^ pancreas,^12,33^ brain,^12,13,14,26,32^ kidney,^30^ and lymph nodes.^29,34^ Furthermore, animal studies demonstrated that rotavirus can penetrate host cells and replicate in nonenterocytes, possibly evading immune responses and systemic sequelae,^35^ indicating potential associations with conditions like type 1 diabetes,^36^ vasculitis,^37^ and neurologic disorders.^38^

The findings of our study provide important insights into the possible etiological relationship of rotavirus infection and autoimmune diseases. In addition, the greater incidence of autoimmune disease in our exposed group (73.1 vs 58.0 per 10 000 person-years, respectively) suggests the importance of awareness for children who experience rotavirus infections at an early age. Overall, our finding suggests that rotavirus-associated hospitalization is associated with increased risk for developing autoimmune diseases that affect different organs and for developing multiple autoimmune syndromes. Previous studies examined the possible mechanism by which rotavirus infection triggered autoimmune processes, and proposed several possible explanations. Although some of the findings of these previous studies are inconsistent and this issue remains unresolved,^39^ studies of antigenic mimicry suggest that humoral and cellular immune responses may be responsible for the autoimmunity reported in studies of humans, animals, and in vitro models.^36,40,41,42^ Gastrointestinal infections from rotavirus can trigger immune responses distinct from those caused by food antigens because of oral tolerance. Gastrointestinal induction of autoimmune responses may disrupt oral tolerance and cause a type 1 T helper cell immune response in predisposed individuals. The high similarity of rotavirus surface proteins known to have gastrointestinal pathogenicity (eg, VP4 and VP7) with bovine milk, casein, and human T-cell epitopes may contribute to increased autoimmunity.^22,36^ A recent study proposed the bystander activation model to explain autoimmune provocation following rotavirus infection,^1^ suggesting that inflammation during the infection reduces self-tolerance and activates autoimmune cells. Higher antibiotic usage was observed in the exposed group, which is also a considered as a potential risk factor for childhood autoimmune diseases.^43^

The strengths of our study are that we used a population-based cohort, complete follow-up of nearly 50 000 patients with a rotavirus-associated hospitalization for nearly 10 years, and a large sample size enabling subgroup analysis and sensitivity analysis. We also considered age at exposure and multiple outcome measures. Detailed sociodemographic and medical data enabled effective control for numerous potential confounders.

### Limitations

This study has limitations. For example, there may have been some surveillance bias. To address this issue, we performed sensitivity analyses that considered extended lag times, different definitions for exposure and outcome, and adjusted for estimated levels of medical surveillance. Additionally, we relied on registered data, which may have led to underestimating cases if infection occurred as a comorbidity, if there were delays in pathogen identification, or if there were missed diagnoses. Furthermore, because this was an observational study, we cannot make causal inferences regarding the relationship of rotavirus-associated hospitalization with autoimmune disease. The potential links of other unmeasured factors, such as unreported health conditions, alterations in health-related behaviors, and the use of certain medications, should be addressed in future studies. Moreover, we were not able assess the association between rotavirus-associated hospitalization with autoimmune diseases later in life.

In addition, in the present data set, we were unable to assess whether the matched unexposed group experienced hospitalization due to other causes. Future studies investigating the underlying contributing factors are warranted.

## Conclusions

In this nationwide cohort study, rotavirus-associated hospitalization was associated with an increased risk for childhood autoimmune disease overall and multiple specific autoimmune diseases. Clinicians should be aware of the increased predisposition for autoimmune disease in individuals who experienced rotavirus-associated hospitalization. Further studies are needed to understand the underlying mechanisms.

## Notes

Supplement 1. **eTable 1.** Human Studies and Case Reports of Rotavirus Infection and Autoimmune Disease **eTable 2.** Nonhuman Studies of Rotavirus Infection and Autoimmune Disease **eTable 3.** MeSH Terms and *ICD-10* Codes Used to Define Exposures and Outcomes **eTable 4.** Terms Used for Searching PubMed **eTable 5.** Risk of Autoimmune Diseases in Participants With and Without Rotavirus Infections Based on Different Indicators of Infection **eTable 6.** Risk of Autoimmune Disease Among Patients With Rotavirus Infection by Length and Number of Hospitalizations **eFigure.** Kaplan-Meier Curve Demonstrating the Cumulative Incidence of Autoimmune Diseases
eReferences.

Supplement 2.
Data Sharing Statement
