# Supplementary material for: Rotavirus-Associated Hospitalization in Children With Subsequent Autoimmune Disease
Source: JAMA Netw Open. 2023 Jul 26;6(7):e2324532. doi: 10.1001/jamanetworkopen.2023.24532 (PMC10372702; doi:10.1001/jamanetworkopen.2023.24532)
Supplement: Supplement 1. — eTable 1. Human Studies and Case Reports of Rotavirus Infection and Autoimmune Disease eTable 2. Nonhuman Studies of Rotavirus Infection and Autoimmune Disease eTable 3. MeSH Terms and ICD-10 Codes Used to Define Exposures and Outcomes eTable 4. Terms Used for Searching PubMed eTable 5. Risk of Autoimmune Diseases in Participants With and Without Rotavirus Infections Based on Different Indicators of Infection eTable 6. Risk of Autoimmune Disease Among Patients With Rotavirus Infection by Length and Number of Hospitalizations eFigure. Kaplan-Meier Curve Demonstrating the Cumulative Incidence of Autoimmune Diseases eReferences. [file jamanetwopen-e2324532-s001.pdf]

Supplemental Online Content

Ha EK, Kim JH, Cha HR, et al. Rotavirus-associated hospitalization in children with subsequent autoimmune disease. *JAMA Netw Open*. 2023;6(7):e2324532. doi:10.1001/jamanetworkopen.2023.24532

eTable 1. Human Studies and Case Reports of Rotavirus Infection and Autoimmune Disease

eTable 2. Nonhuman Studies of Rotavirus Infection and Autoimmune Disease

eTable 3. MeSH Terms and *ICD-10* Codes Used to Define Exposures and Outcomes

eTable 4. Terms Used for Searching PubMed

eTable 5. Risk of Autoimmune Diseases in Participants With and Without Rotavirus Infections Based on Different Indicators of Infection

eTable 6. Risk of Autoimmune Disease Among Patients With Rotavirus Infection by Length and Number of Hospitalizations

eFigure. Kaplan-Meier Curve Demonstrating the Cumulative Incidence of Autoimmune Diseases

eReferences.

This supplemental material has been provided by the authors to give readers additional information about their work.

© 2023 Ha EK et al. *JAMA Network Open.*

eTable 1. Human studies and Case reports of *Rotavirus* infection and Autoimmune Disease

Autoimmune disease	Population	Study design	Published year	Results	Author (reference)
Hemolytic Uremic Syndrome	Indian	Hospital-based surveillance 2012–2015	2018	Higher prevalence of GI pathogens in auto-immune HUS	Togarsimalemath SK[1]
Nonceliac gluten sensitivity (NCGS)	Italy	Hospital-based surveillance	2018	Possible involvement of *Rotavirus* infection in the pathogenesis of NCGS	Puccetti A[2]
Celiac Disease	United States and Europe	Hospital-based surveillance 2005–2015	2017	Gastrointestinal infections increase the risk of celiac disease autoimmunity in children with genetic susceptibility to this autoimmune disorder.	Kemppainen KM[3]
Celiac Disease	Italy	Hospital-based surveillance 2014–2015	2016	Children with CD do not have higher immune reactivity to RV	Ziberna F[4]
Pemphigus Vulgaris	United States	Hospital-based surveillance, years not mentioned	2016	Certain VH1-46 (B cells reacting in pemphigus vulgaris) B cell populations may be predisposed to Dsg3-VP6 cross-reactivity, but multiple mechanisms prevent the onset of autoimmunity after *Rotavirus* exposure.	Cho MJ[5]
Type 1 Diabetes	United States	Hospital-based surveillance, years not mentioned	2015	Negative role for *Rotavirus* in Type 1 Diabetes	Bian X[6]
Acute encephalopathy associated with hemolytic uremic syndrome	Japan	Case report	2015	Involvements of verotoxin 1 and 2 caused by O157: the H7 strain of the enterohemorrhagic Escherichia coli and *Rotavirus* were presumed	Imataka G[7]
Opsoclonus-myoclonus syndrome	Turkey	Case report	2014	Opsoclonus–myoclonus syndrome seems to be a CNS complication of *Rotavirus* infection	Gurkas E[8]
Type 1 Diabetes	Israel	Hospital-based surveillance 2010	2014	Support the hypothesis that maternal *Rotavirus* infections during pregnancy may damage the fetal islet cells and trigger the cascade of events leading to Type 1 diabetes	Shulman LM[9]

© 2023 Ha EK et al. *JAMA Network Open.*

Celiac disease	Italy	Hospital-based surveillance 2010	2013	Support the link between *Rotavirus* infection and CD	Dolcino M[10]
Kawasaki disease	United States and Denmark	Hospital-based and National data	2012	Suspected infectious etiology	Pitzer VE[11]
Inflammatory Bowel Disease	Finland	Hospital-based surveillance 2010	2012	Enteral viruses play a minor role in the disease activity of pediatric IBD	Kolho KL[12]
Type 1 Diabetes	Finland	Hospital-based surveillance 2010	2012	Failed to find any association	Lempainen J[13]
Guillain-Barré syndrome	Japan	Case report	2011	*Rotavirus* antigens assume to shed outside of the intestines during the acute phase of the illness and might have bound to asialo-GM1 in myelin	Kamihiro N[14]
Celiac disease	Iran	Population-based surveillance 2006–2007	2010	Prevalence of active RV infection was not statistically significantly different between individuals who were tTG antibody positive and those who were tTG antibody negative	Rostami-Nejad M[15]
Immune thrombocytopenic purpura	United States	Case report	2010	Proposed that the development of ITP in this infant might have been associated with *Rotavirus* s infection	Siddiqui AH[16]
Kawasaki disease	United States	Hospital-based surveillance 2000–2005	2009	Cannot completely rule out rotavirus as having a role in the development of KS, other agents must be involved in the etiology of KS	MacNeil A[17]
Gluten sensitivity	Italy	Case report	2007	Positive GS-associated autoantibodies and small intestinal biopsies) months after gastrointestinal infection due to *Rotavirus*	Pavone P[15]
Type 1 Diabetes	Finland	Population-based birth cohort	2006	Did not provide evidence supporting an association between *Rotavirus* infections and the development of type 1 diabetes or diabetes-associated autoantibodies in young children	Mäkelä M[18]
Celiac disease	United States	Hospital-based surveillance cohort	2006	High frequency of *Rotavirus* infections may increase the risk of celiac disease autoimmunity in childhood in genetically predisposed individuals	Stene LC[19]
Acute necrotizing encephalopathy	Canada	Case report	2005	Cases of acute necrotizing encephalopathy including unique aspects association with	Kirton A[20]

© 2023 Ha EK et al. *JAMA Network Open.*

				varicella and *Rotavirus* infection	
Type 1 Diabetes	Finland	Hospital-based surveillance	2002	*Rotavirus* infections are unlikely triggers of beta-cell autoimmunity in young children with genetic susceptibility to type 1 diabetes.	Blomqvist M[21]
Type 1 Diabetes	Australia	Hospital-based surveillance	2000	Associated with temporary increases in the levels of T1D-associated autoantibodies	Honeyman MC[22]
Guillain-Barré syndrome	The Netherlands	Case report	1999	GBS in a very young infant after *Rotavirus* related gastroenteritis	Smeets CC
Type 1 Diabetes	Australia	Hospital-based	1998	Identified T-cell epitope peptides in the intracytoplasmic domain of the type 1 diabetes autoantigen, tyrosine phosphatase IA-2, whose sequence analysis suggests that immunity to *Rotavirus*	Honeyman MC[24]
Pancreatitis and hypoglycemia-associated convulsions	Italy	Case report	1991	Pancreatitis and islet-cell antibodies have been associated with *Rotavirus* infection	Nigro G

© 2023 Ha EK et al. *JAMA Network Open.*

eTable 2. Non-human studies of *Rotavirus* infection and Autoimmune Disease

Autoimmune disease	Study design	Published year	Results	Author (reference)
Transient Pancreatic Involution and Hyperglycemia	*in vivo* animal study (mice)	2014	Demonstrated pro-apoptotic effect of rhesus Rotavirus on the pancreas	Honeyman MC[26]
Myasthenia gravis	In silico	2014	Detected rotaviral VP6 as a potential threat for myasthenia gravis and enlighten an area of virus associated autoimmune research.	Sarkar T[27]
Type 1 Diabetes	Animal study (mice)	2014	Support a possible role for bystander activation in type 1 diabetes acceleration by Rhesus monkey rotavirus	Pane JA[28]
Autoimmune uveitis	in vitro and in vivo animal study (Lewis rats)	2003	Oral tolerization with retinal S-antigen (PDSAg), but not with rotavirus- and casein-derived peptides or caseinprotein, prevented PDSAg-induced uveitis in rats.	Wildner G[29]

© 2023 Ha EK et al. *JAMA Network Open.*

eTable 3. MeSH terms and ICD-10 codes used to define exposures and outcomes

Disease		MeSH Term	ICD-10 code
Rotavirus infection	Rotavirus enteritis	" Rotavirus *"[All Fields]	A08.0x
Disease of endocrine system	Diabetes mellitus, insulin dependent	Diabetes mellitus, type 1	E10
	Autoimmune thyroid disease	Thyroiditis, autoimmune	E03.5, E03.9, E05.0, E05.5, E05.9, E06.3, E06.5
	Addison's disease	Addison Disease	E27.1, E27.2
	Autoimmune polyglandular syndrome	Polyendocrinopathies, autoimmune; Autoimmune polyendocrinopathy syndrome type 1	E31.0
Inflammatory arthritis	Reactive arthritis (Reiter syndrome)	Arthritis, reactive	M02.3, M02.8, M02.9, M05, M06, M08.0
	Rheumatoid arthritis	Arthritis, juvenile	M08.1, M08.2, M08.3. M08.4
	Ankylosing spondylitis	Spondylitis, ankylosing	M45
Vasculitis	Kawasaki disease and related condition	Mucocutaneous Lymph Node Syndrome; Polyarteritis Nodosa; Churg-Strauss Syndrome	M30
	Thrombotic microangiopathy	Thrombotic Microangiopathies	M31.1
	Wegener's granulomatosis	Granulomatosis with Polyangiitis	M31.3
	Microscopic polyangiitis	Microscopic Polyangiitis	M31.7
	Henoch-Schonlein purpura	Vascular purpura	D69.0
	Giant cell arteritis/polymyalgia rheumatica	Giant Cell Arteritis; Polymyalgia Rheumatica	M35.3, M31.5, M31.6
Connective tissue disorders	Systemic lupus erythematosus	Lupus erythematosus, systemic	M32
	Polymyositis/dermatomyositis	Polymyositis; Dermatomyositis	M33.0, M33.1, M33.2, M33.9
	Systematic sclerosis (scleroderma)	Scleroderma, systemic; Scleroderma, diffuse	M34
	Sjögren's syndrome	Sjogren's Syndrome	M35.0
	Mixed connective tissue disease	Mixed Connective Tissue Disease	M35.1

© 2023 Ha EK et al. *JAMA Network Open.*

	Behcet’s syndrome	Behcet Syndrome	M35.2
Disease of skin system	Pemphigus vulgaris	Pemphigus	L10.0
	Bullous pemphigoid	Pemphigoid, bullous	L12
	Dermatitis herpetiformis	Dermatitis Herpetiformis	L13.0
	Psoriasis	Psoriasis	L40
	Alopecia areata	Alopecia Areata	L64
	Vitiligo	Vitiligo	L80
Hematological diseases	Pernicious anemia	Anemia, pernicious	D51.0
	Autoimmune hemolytic anemia	Anemia, hemolytic, autoimmune	D59.0, D59.1
	Idiopathic thrombocytopenic purpura	Purpura, thrombocytopenic, idiopathic	D69.3
Disease of nervous system	Acute disseminated encephalomyelitis	Encephalomyelitis, acute disseminated	G04
	Anti-NMDA receptor encephalitis	Anti-N-Methyl-D-Aspartate Receptor Encephalitis	G13.1
	Multiple sclerosis	Multiple Sclerosis; Multiple sclerosis, chronic progressive; Multiple sclerosis, relapsing remitting	G35
	Neuromyelitis optica and ADEM	Neuromyelitis Optica	G36
	Guillain-Barré syndrome	Guillain-Barre Syndrome	G61.0, G61.1, G61.8, G61.9
	Myasthenia gravis	Myasthenia Gravis	G70.0
Disease of digestive system	Primary biliary cirrhosis	Liver cirrhosis, biliary	K74.3
	Crohn’s disease	Crohn Disease	K50
	Ulcerative colitis	Colitis, ulcerative	K51
	Coeliac disease	Celiac Disease	K90.0
Others	Acute rheumatic fever and chorea	Rheumatic Fever; Chorea	I00, I01.0, I01.1, I01.2, I01.8, I01.9, I02.0, I02.9
	Sarcoidosis	Sarcoidosis; Sarcoidosis, pulmonary	D86
	IgA nephropathy	Glomerulonephritis, IgA	N00,N01,N03,N05

Abbreviations: ADEM, acute disseminated encephalomyelitis; ICD, International Classification of Diseases; NMDA, N-Methyl-D-aspartate.

© 2023 Ha EK et al. *JAMA Network Open.*

eTable 4. Terms used for searching PubMed

1	"Rotavirus"[Mesh]	12,357
2	Rotavirus infections *	212,511
3	("Rotavirus"[Mesh]) OR (Rotavirus infections*)	212,511
4	MESH terms for Autoimmune disease (See ETable 2)	
5	Mycoplasma AND Autoimmune disease	

© 2023 Ha EK et al. *JAMA Network Open.*

eTable 5. Risk of autoimmune diseases in participants with and without *Rotavirus* infections based on different indicators of infection

Rotavirus infection indicators	Patients with *Rotavirus* at admission				
	No. of autoimmune disease cases/No. of accumulated person-years 10,000 (incidence rate/10,000 person-years)		Absolute rate difference/10,000 person-years (95% CI) [a]	Hazard ratio (95% CI)[b]	*P* value[c]
	Exposed individuals	Matched unexposed individuals			
Use of antibiotics [d,e]					
No	290/40.2 (7.2)	1,623/302.6 (5.4)	18.50 (9.80–27.21)	1.27 (1.12-1.45)	0.30
Yes	7,299/998.2 (7.3)	4,473/748.4 (6.0)	13.37 (10.94–15.79)	1.20 (1.16-1.25)	
Use of systemic corticosteroid [d,f]					
No	5,312/731.1 (7.3)	4,909/855.8 (5.7)	15.30 (12.77–17.82)	1.25 (1.20-1.30)	0.60
Yes	2,277/307.3 (7.4)	1,186/195.3 (6.1)	13.37 (8.76–17.98)	1.19 (1.10-1.27)	
Frequency of outpatient visits within a month after index date[g]					
No	133 / 20.8 (6.4)	970/193.5 (5.0)	13.81 (2.50–25.13)	1.12 (0.92–1.37)	0.78
Yes	7,456/1017.6 (7.3)	5,125/857.5 (6.0)	13.50 (11.17–15.84)	1.21 (1.16-1.25)	

[a] Results were not calculated if the number of cases was fewer than 10.

[b] Cox models were stratified by matching identifiers (birth year and sex) and adjusted for birth residence (Seoul/metropolitan, city, or rural area), household income (low, middle, or high), perinatal history (disorders related to fetal growth and development, birth injury, congenital malformations), and prescription duration of medications (macrolide antibiotics and systemic steroids). The first year of follow-up was excluded from all analyses.

[c] *P* value was from an interaction test, by incorporating an interaction term to the Cox model.

[d] Prescription from 1 month before to 1 month after the index date.

[e] The median prescription duration was 18 days.

[f] The median prescription duration was 4 days.

[g] The median outpatient visit event was 3 days.

© 2023 Ha EK et al. *JAMA Network Open.*

eTable 6. Risk of Autoimmune Disease among Patients with *Rotavirus* infection by length and number of hospitalizations.

	**Patients admitted with *Rotavirus* infection[a]**			
	Cases/accumulated person-years × 10,000 (Incidence rate/10,000 person-years)	Absolute Rate Difference/10,000 Person-Years (95% CI)	Hazard Ratio (95% CI)[b]	*P* Value[c]
Length of hospitalization[d]				**0.0404**
No hospitalization[e]	6,095/ 86,157 (58.0)			
≤5 days	3,700/45,400 (69.0)	10.97 (8.31-13.62)	1.17 (1.12-1.22)	
>5 days	3,889/40,757 (77.5)	19.51 (16.68-22.35)	1.31 (1.26-1.37)	
Number of hospitalization event				**<0.001**
No event	6,095/86,157 (58.0)			
Single event	6,707/78,653 (70.9)	12.91 (10.68-15.15)	1.20 (1.16-1.24)	
Multiple events	882/7,504 (95.5)	37.57 (31.10-44.04)	1.60 (1.49-1.72)	

[a] Results not shown if the number of subjects was less than 10.

[b] Cox models were stratified by matching identifiers (birth year and sex) and adjusted for birth residence (Seoul/metropolitan, city, or rural area), household income (low, middle, or high), and perinatal history (disorder related to length of gestation and fetal growth, birth trauma, infections specific to the perinatal period, congenital malformation/deformation, and chromosomal abnormalities). The first year of follow-up was excluded from all analyses.

[c] *P* value was from comparison test of HRs within subgroups calculated by using the difference in the log HR between strata/

[d] The median length of hospitalization was 5 days.

[e] Indicates the matched unexposed individuals.

© 2023 Ha EK et al. *JAMA Network Open.*

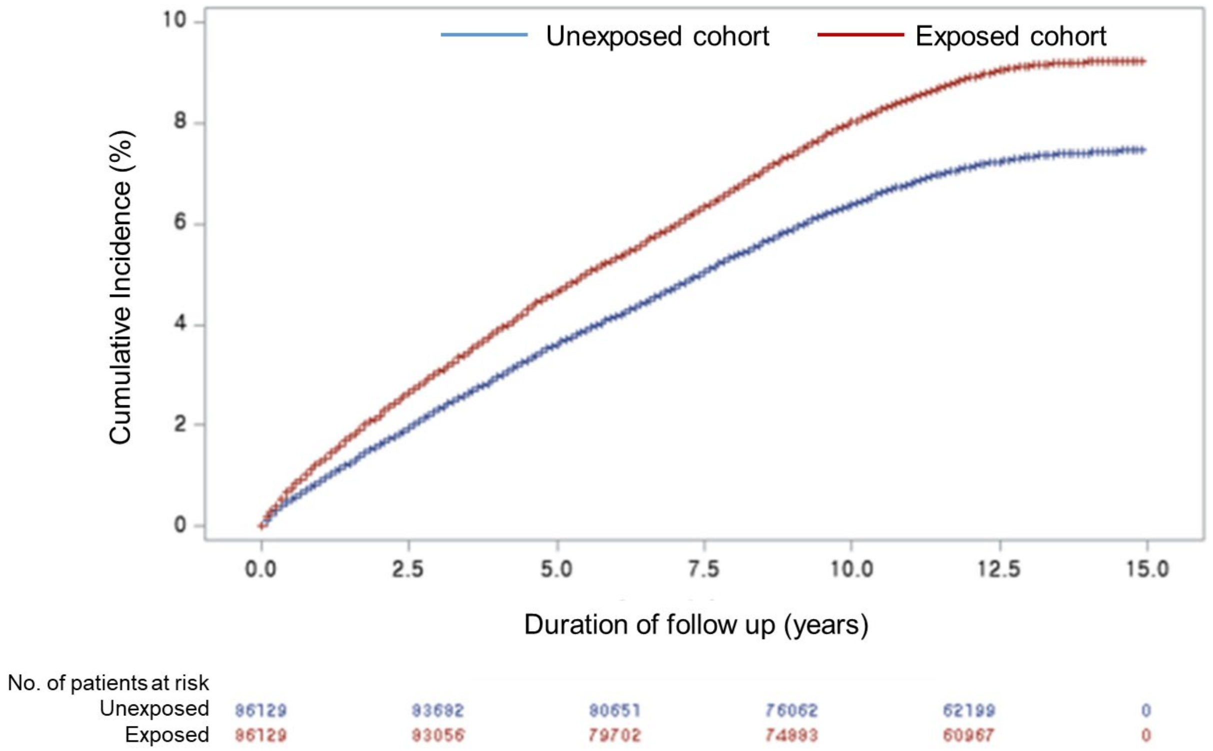

eFigure. Kaplan-Meier curve demonstrating the cumulative incidence of autoimmune diseases[a]. The red line shows the cumulative incidence for outcome events in the exposed cohort and the blue line in the unexposed cohort.

[a] The first year of follow-up was considered as a window period and excluded from calculation for cumulative incidence rates of autoimmune disease. The duration of follow up was calculated from the period after the window period.

© 2023 Ha EK et al. *JAMA Network Open.*

eReferences

1. Togarsimalemath SK, Si-Mohammed A, Puraswani M, et al. Gastrointestinal pathogens in anti-FH antibody positive and negative Hemolytic Uremic Syndrome. Pediatr Res. 2018;84(1):118-24.
2. Puccetti A, Saverino D, Opri R, et al. Immune Response to Rotavirus and Gluten Sensitivity. J Immunol Res. 2018;2018:9419204.
3. Kemppainen KM, Lynch KF, Liu E, et al. Factors That Increase Risk of Celiac Disease Autoimmunity After a Gastrointestinal Infection in Early Life. Clin Gastroenterol Hepatol. 2017;15(5):694-702 e5.
4. Ziberna F, De Lorenzo G, Schiavon V, et al. Lack of evidence of rotavirus-dependent molecular mimicry as a trigger of coeliac disease. Clin Exp Immunol. 2016;186(3):356-63.
5. Cho MJ, Ellebrecht CT, Hammers CM, et al. Determinants of VH1-46 Cross-Reactivity to Pemphigus Vulgaris Autoantigen Desmoglein 3 and Rotavirus Antigen VP6. J Immunol. 2016;197(4):1065-73.
6. Bian X, Wallstrom G, Davis A, et al. Immunoproteomic Profiling of Antiviral Antibodies in New-Onset Type 1 Diabetes Using Protein Arrays. Diabetes. 2016;65(1):285-96.
7. Imataka G, Wake K, Suzuki M, Yamanouchi H, Arisaka O. Acute encephalopathy associated with hemolytic uremic syndrome caused by Escherichia coli O157: H7 and rotavirus infection. Eur Rev Med Pharmacol Sci. 2015;19(10):1842-4.
8. Gurkas E, Gucuyener K, Yilmaz U, Havali C, Demir E. Opsoclonus-myoclonus syndrome following rotavirus gastroenteritis. Pediatr Int. 2014;56(6):e86-e7.
9. Shulman LM, Hampe CS, Ben-Haroush A, et al. Antibodies to islet cell autoantigens, rotaviruses and/or enteroviruses in cord blood and healthy mothers in relation to the 2010-2011 winter viral seasons in Israel: a pilot study. Diabet Med. 2014;31(6):681-5.
10. Dolcino M, Zanoni G, Bason C, et al. A subset of anti-rotavirus antibodies directed against the viral protein VP7 predicts the onset of celiac disease and induces typical features of the disease in the intestinal epithelial cell line T84. Immunol Res. 2013;56(2-3):465-76.
11. Pitzer VE, Burgner D, Viboud C, et al. Modelling seasonal variations in the age and incidence of Kawasaki disease to explore possible infectious aetiologies. Proc Biol Sci. 2012;279(1739):2736-43.
12. Kolho KL, Klemola P, Simonen-Tikka ML, Ollonen ML, Roivainen M. Enteric viral pathogens in children with inflammatory bowel disease. J Med Virol. 2012;84(2):345-7.
13. Lempainen J, Tauriainen S, Vaarala O, et al. Interaction of enterovirus infection and cow's milk-based formula nutrition in type 1 diabetes-associated autoimmunity. Diabetes Metab Res Rev. 2012;28(2):177-85.
14. Kamihiro N, Higashigawa M, Yamamoto T, et al. Acute motor-sensory axonal Guillain-Barre syndrome with unilateral facial nerve paralysis after rotavirus gastroenteritis in a 2-year-old boy. J Infect Chemother. 2012;18(1):119-23.
15. Rostami-Nejad M, Rostami K, Sanaei M, et al. Rotavirus and coeliac autoimmunity among adults with non-specific gastrointestinal symptoms. Saudi Med J. 2010;31(8):891-4.
16. Siddiqui AH, Chitlur MB. Immune thrombocytopenic purpura in a 5-month-old female with rotavirus infection. Pediatr Blood Cancer. 2010;54(4):633.
17. MacNeil A, Holman RC, Yorita KL, Steiner CA, Parashar UD, Belay ED. Evaluation of seasonal patterns of Kawasaki syndrome- and rotavirus-associated hospitalizations in California and New York, 2000-2005. BMC Pediatr. 2009;9:65.
18. Makela M, Oling V, Marttila J, et al. Rotavirus-specific T cell responses and cytokine mRNA expression in children with diabetes-associated autoantibodies and type 1 diabetes. Clin Exp Immunol. 2006;145(2):261-70.
19. Stene LC, Honeyman MC, Hoffenberg EJ, et al. Rotavirus infection frequency and risk of celiac disease autoimmunity in early childhood: a longitudinal study. Am J Gastroenterol. 2006;101(10):2333-40.
20. Kirton A, Busche K, Ross C, Wirrell E. Acute necrotizing encephalopathy in caucasian children: two cases and review of the literature. J Child Neurol. 2005;20(6):527-32.
21. Blomqvist M, Juhela S, Erkkila S, et al. Rotavirus infections and development of diabetes-associated autoantibodies during the first 2 years of life. Clin Exp Immunol. 2002;128(3):511-5.
22. Honeyman MC, Coulson BS, Stone NL, et al. Association between rotavirus infection and pancreatic islet autoimmunity in children at risk of developing type 1 diabetes. Diabetes. 2000;49(8):1319-24.
23. Smeets CC, Brussel W, Leyten QH, Brus F. First report of Guillain-Barre syndrome after rotavirus-induced gastroenteritis in a very young infant. Eur J Pediatr. 2000;159(3):224.
24. Honeyman MC, Stone NL, Harrison LC. T-cell epitopes in type 1 diabetes autoantigen tyrosine phosphatase IA-2: potential for mimicry with rotavirus and other environmental agents. Mol Med. 1998;4(4):231-9.
25. Nigro G. Pancreatitis with hypoglycemia-associated convulsions following rotavirus gastroenteritis. J Pediatr Gastroenterol Nutr. 1991;12(2):280-2.

© 2023 Ha EK et al. *JAMA Network Open.*

26. Honeyman MC, Laine D, Zhan Y, Londrigan S, Kirkwood C, Harrison LC. Rotavirus infection induces transient pancreatic involution and hyperglycemia in weanling mice. PLoS One. 2014;9(9):e106560.
27. Sarkar T, Das S, Nandy P, Bhowmick R, Nandy A. In silico study of potential autoimmune threats from rotavirus infection. Comput Biol Chem. 2014;51:51-6.
28. Pane JA, Webster NL, Zufferey C, Coulson BS. Rotavirus acceleration of murine type 1 diabetes is associated with increased MHC class I-restricted antigen presentation by B cells and elevated proinflammatory cytokine expression by T cells. Virus Res. 2014;179:73-84.
29. Wildner G, Diedrichs-Mohring M. Autoimmune uveitis induced by molecular mimicry of peptides from rotavirus, bovine casein and retinal S-antigen. Eur J Immunol. 2003;33(9):2577-87.

© 2023 Ha EK et al. *JAMA Network Open.*
