# Supplementary material for: Rotavirus-Associated Hospitalization in Children With Subsequent Autoimmune Disease
Source: JAMA Netw Open. 2023 Jul 26;6(7):e2324532. doi: 10.1001/jamanetworkopen.2023.24532 (PMC10372702; doi:10.1001/jamanetworkopen.2023.24532)
Supplement: Supplement 2. — Data Sharing Statement [file jamanetwopen-e2324532-s002.pdf]

Data Sharing Statement

Ha. Rotavirus-Associated Hospitalization in Children With Subsequent Autoimmune Disease. *JAMA Netw Open*. Published July 26, 2023. doi:10.1001/jamanetworkopen.2023.24532

Data
Data available: Data is available from the author(s) with the permission of the National Health Insurance Service (NHIS). Restrictions apply to the availability of these data used under license for this study.
